# Supplementary figures and images for: Age-Dependent Redistribution of the Life-Important Enzyme in the Retina: Adult Müller Glial Cells’ Endfeet Lack Spermine Synthase Expression
Source: Biomolecules. 2025 Sep 27;15(10):1374. doi: 10.3390/biom15101374 (PMC12563582; doi:10.3390/biom15101374)

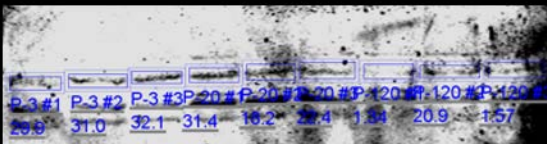

$\beta$ -actin

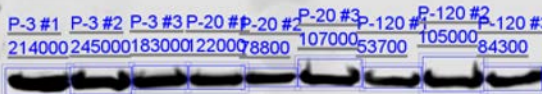

Supplement: Supplementary file 1 [file biomolecules-15-01374-s001.zip › biomolecules-3714562-supplementary.pdf]
